# Alterations in white matter microstructure in alcohol and alcohol‐polydrug dependence: Associations with lifetime alcohol and nicotine exposure

**DOI:** 10.1111/adb.13207

**Published:** 2022-07-18

**Authors:** Kofoworola Agunbiade, Leon Fonville, John McGonigle, Rebecca Elliott, Karen D. Ersche, Remy Flechais, Csaba Orban, Anna Murphy, Dana G. Smith, John Suckling, Eleanor M. Taylor, Bill Deakin, Trevor W. Robbins, David J. Nutt, Anne R. Lingford‐Hughes, Louise M. Paterson, ICCAM Consortium, David Nutt, Anne Lingford‐Hughes, Louise Paterson, John McGonigle, Remy Flechais, Csaba Orban, Bill Deakin, Rebecca Elliott, Anna Murphy, Eleanor Taylor, Trevor Robbins, Karen Ersche, John Suckling, Dana Smith, Laurence Reed, Filippo Passetti, Luca Faravelli, David Erritzoe, Inge Mick, Nicola Kalk, Adam Waldman, Liam Nestor, Shankar Kuchibatla, Venkataramana Boyapati, Antonio Metastasio, Yetunde Faluyi, Emilio Fernandez‐Egea, Sanja Abbott, Barbara Sahakian, Valerie Voon, Ilan Rabiner

**Affiliations:** ^1^ Division of Psychiatry, Department of Brain Sciences Imperial College London London UK; ^2^ Neuroscience and Psychiatry Unit, Institute of Brain, Behaviour and Mental Health University of Manchester Manchester UK; ^3^ Behavioural and Clinical Neuroscience Institute University of Cambridge Cambridge UK; ^4^ Department of Psychiatry University of Cambridge Cambridge UK; ^5^ Department of Systems Neuroscience University Medical Centre Hamburg‐Eppendorf Hamburg Germany; ^6^ Department of Psychology University of Cambridge Cambridge UK

**Keywords:** alcohol dependence, polydrug dependence, addiction, neuroimaging, DTI, TBSS

## Abstract

Evidence suggests that alcohol dependence (AD) is associated with microstructural deficits in white matter, but the relationship with lifetime alcohol exposure and the impact of polydrug dependence is not well understood. Using diffusion tensor magnetic resonance (MR) imaging, we examined white matter microstructure in relation to alcohol and polydrug dependence using data from the Imperial College Cambridge Manchester (ICCAM) platform study. Tract‐based spatial statistics were used to examine fractional anisotropy (FA) in a cohort of abstinent AD participants, most of whom had a lifetime history of dependence to nicotine. A further subgroup also had a lifetime history of dependence to cocaine and/or opiates. Individuals with AD had lower FA throughout the corpus callosum, and negative associations with alcohol and nicotine exposure were found. A group‐by‐age interaction effect was found showing greater reductions with age in the alcohol‐dependent group within corpus callosum, overlapping with the group difference. We found no evidence of recovery with abstinence. A comparison of alcohol‐only‐ and alcohol‐polydrug‐dependent groups found no differences in FA. Overall, our findings show that AD is associated with lower FA and suggest that these alterations are primarily driven by lifetime alcohol consumption and cigarette smoking, showing no relationship with exposure to other substances such as cocaine, opiates or cannabis. Reductions in FA across the adult lifespan are more pronounced in AD and offer further support for the notion of accelerated ageing in relation to alcohol dependence. These findings highlight there may be lasting structural differences in white matter in alcohol dependence, despite continued abstinence.

## Graphical abstract

Alcohol‐dependent participants showed lower fractional anisotropy (FA) within the corpus callosum compared with healthy controls. This change was negatively associated with lifetime alcohol and nicotine exposure, and there was no evidence of FA recovery despite prolonged abstinence. A group‐by‐age interaction was observed, whereby greater age‐related reductions in FA were evident in AD participants, but no further reductions in FA were observed in alcohol‐polysubstance relative to alcohol‐only‐dependent participants.

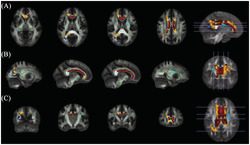

## 1 INTRODUCTION

Alcohol dependence (AD) is characterised by a preoccupation with seeking alcohol, loss of control over use and continued drinking in spite of harms and is associated with high rates of relapse. An important clinical priority is the identification of biomarkers that predict different stages of disease projection, for example, progression from ‘heavy use’ to dependence or vulnerability to relapse. Increased understanding of the role of white matter in addiction will likely lead to improvements in prediction of substance dependence, as white matter is a critical structure that facilitates connections between regions of the cortex. An initial meta‐analytic investigation of white matter macrostructure in AD revealed a modest general reduction in volume relative to healthy controls.^1^ Subsequently, a recent coordinate‐based meta‐analysis found common volume reductions in the anterior thalamic radiation in alcohol, nicotine and cocaine dependence.^2^ Diffusion tensor imaging (DTI) allows for a more detailed assessment of white matter and to elucidate the relationship between structural connectivity and substance dependence. Unsurprisingly, DTI studies in AD have typically found differences in white matter microstructure, with the vast majority reporting a negative impact on white matter tracts.^3^

One of the most widely reported diffusion metrics is fractional anisotropy (FA). Tract‐based spatial statistics (TBSS) allows for a voxel‐based approach to examine local white matter microstructure using diffusion indices, typically using whole‐brain maps of FA.^4^ TBSS studies of AD have frequently reported lower FA in the corpus callosum,^5^, ^6^, ^7^, ^8^, ^9^, ^10^, ^11^, ^12^ with mixed findings implicating other tracts and regions. A meta‐analysis of white matter macro‐ and microstructural differences in AD further confirmed that alterations converge on the corpus callosum, cingulum bundle and internal capsule.^13^ Considering only the 11 identified DTI studies, differences were solely found along the corpus callosum and fornix. It is important to note that though most studies have examined recently abstinent patients,^5^, ^6^, ^7^, ^8^, ^9^, ^10^ similar disturbances in the corpus callosum have been found in long‐term abstinent^8^ and non‐treatment‐seeking patients.^11^ This could highlight a static and long‐term impact of AD on white matter microstructure. However, longitudinal studies have pointed to an attenuation of differences with abstinence^14^, ^15^, ^16^, ^17^ as well as greater deterioration in those who relapse to heavy drinking.^15^, ^18^ This implies that deficits arise as a result of chronic excessive alcohol consumption, in support with animal^10^ and post‐mortem neuropathology^19^ studies, and are amenable to recovery. Additionally, it has been suggested that some microstructural deficits may reflect a pre‐existing vulnerability to development of AD.^20^, ^21^

Studies seeking to distinguish subtypes of AD have tended to include a group characterised by a high occurrence of other forms of substance abuse and dependence.^22^, ^23^, ^24^ Polydrug use by clients within treatment services has been long been recognised as a treatment issue, though this remains understudied, with inpatient prevalence rates of alcohol‐polydrug dependence ranging from 47% to 64%.^25^, ^26^, ^27^ Furthermore, emerging evidence has highlighted worse treatment outcomes—greater risk of relapse, lower treatment retention rates, higher mortality rates and a greater prevalence of psychiatric comorbidity.^28^ A recent meta‐analysis of grey matter volume in substance dependence highlighted that whereas AD is primarily associated with lower volume in frontal and anterior cingulate regions, polydrug users show lower volume in the thalamus and across temporal regions as well as greater volume in the subcallosal extension of the anterior cingulate cortex and putamen.^2^ Direct comparisons of dependent subgroups have been mixed; studies have found lower subcortical volume in alcohol‐only,^29^, ^30^ reduced anterior cingulate thickness and prefrontal surface area,^31^ greater cortical white matter volume in alcohol‐polydrug^32^ and lower cortical white matter volume and lower anterior cingulate white matter in alcohol‐polydrug.^33^ Though there have been no TBSS studies on alcohol‐polydrug dependence, studies have consistently revealed lower FA in cocaine,^34^, ^35^, ^36^ opiate^37^, ^38^ and nicotine dependence^39^, ^40^ and in long‐term cannabis use,^41^, ^42^ particularly in corpus callosum.

This study aimed to replicate previous findings of reduced FA in AD and to investigate the impact of polydrug dependence in a cross‐sectional design. We first compared FA between controls and abstinent AD and then compared those who were alcohol‐only dependent with alcohol‐polydrug dependent and explored associations with measures of substance exposure. Data were derived from the Imperial College Cambridge Manchester (ICCAM) platform,^43^ a multicentre multimodal neuroimaging study that aimed to better understand the neurobiological mechanisms involved in addiction. Participants were healthy controls or abstinent AD individuals with or without additional lifetime cocaine, opiate or other polydrug dependence. We hypothesised that as AD negatively impacts white matter, FA will be lower relative to controls. We expected group differences in FA to be negatively associated with cumulative lifetime alcohol exposure. Alterations to white matter may be amenable with recovery, and we expected FA in affected tracts to be positively associated with abstinence length. Alcohol‐polydrug dependence may lead to additional white matter deficits, and we hypothesise that lower FA will be evident in alcohol‐polydrug compared with alcohol‐only groups.

## 2 METHODS

### 2.1 Participants and study design

Participants were recruited from the ICCAM platform study cohort,^43^ which included 155 participants: healthy controls (*n* = 68), alcohol‐only dependent (*n* = 28) and polydrug (alcohol, cocaine, opiates) dependent (*n* = 59), of which a significant proportion were alcohol‐polydrug dependent (*n* = 36). Participants were included if they met Diagnostic and statistical manual for mental disorders, 4th edition (DSM‐IV) criteria for AD, either alone or in combination with at least one of either cocaine or opiate dependence, with a range of abstinence lengths (0–72 months). Healthy controls did not meet current or past aforementioned dependence criteria and were matched as far as possible with abstinent dependent groups for age, sex and tobacco smoking status. Exclusion criteria included current primary Axis I psychiatric condition, history of severe mental illness, history of psychosis (unless brief and drug‐induced) and history or presence of significant neurological disorders including significant head injury and MRI contraindications. Participants provided a negative breath alcohol and urine drug test prior to scanning (for amphetamines, barbiturates, cocaine, heroin and benzodiazepines). Participants were requested to refrain from cannabis use for at least 7 days, but positive results were permitted due to the long half‐life of metabolites. Additional inclusion and exclusion criteria can be found in Paterson *et al*.^43^ Structural brain scans were assessed by a neuroradiologist in case of adventitious findings. AD participants were followed‐up every 3 months for up to 12 months via telephone interview to obtain self‐reported measures of relapse.

This study was conducted in accordance with the Declaration of Helsinki. Ethical approval was obtained from West London and Gene Therapy Advisory Committee National Research Ethics Service committee (11/H0707/9), and relevant research governance and Participation Identification Centre (PIC) approvals were obtained.

The study consisted of a single baseline scan comprising structural, functional and DTI imaging acquisition protocols and a clinical interview session, during which written informed consent was obtained and eligibility confirmed. Diagnoses were reviewed by a psychiatrist. Substance‐dependent individuals were abstinent at the time of scanning (at least 24 h). The timeline follow‐back method was used to calculate lifetime substance exposure. We defined exposure (alcohol, cocaine, opiates and cannabis) as the number of years in which an individual's consumption could be categorised as ‘heavy use’ for over 6 months within a 12‐month period. Further details on the criteria used to define heavy use and exposure are provided in the Supporting Information.

Three healthy controls were subsequently excluded from analysis: one due to poor quality imaging data, and two were a poor match for the AD group (outliers on age and WTAR IQ scores, i.e. younger with higher IQ than the rest of the cohort). The final groups used for analytical comparison were an alcohol‐dependent cohort (AD, *n* = 64), which was then split into two subgroups of alcohol‐polydrug‐dependent (*n* = 36) and alcohol‐dependent‐only (*n* = 28) individuals and healthy controls (HC, *n* = 65).

### 2.2 MRI acquisition

Images were acquired using a 3‐T Siemens Tim Trio MRI scanner using a 32‐channel headcoil at the Cambridge and Imperial sites and a 3‐T Philips Achieva scanner with eight‐channel headcoil in Manchester. Diffusion‐weighted images were acquired using a single‐shot spin‐echo echo‐planar imaging sequence (TR = 9300 ms, TE = 88 ms, acquisition matrix = 128 × 128, voxel resolution = 1.9 mm^3^) with 64 gradient orientations (b = 1000 s/mm^2^) and one unweighted scan (b = 0 s/mm^2^). Parameters were identical between all three sites, though at the Manchester site, acceleration was achieved using sensitivity encoding (SENSE) instead of generalized autocalibrating partial parallel acquisition (GRAPPA). A high‐resolution T_1_‐weighted image was acquired at each site using a magnetization‐prepared rapid gradient echo (MPRAGE) sequence (Siemens; TR = 2300 ms, TE = 2.98 ms, TI = 900 ms, flip angle = 9°, image matrix = 240 × 256, voxel resolution = 1 mm^3^, Philips; TR = 6.8 ms, TE = 3.1 ms, TI = 900 ms, flip angle = 9°, image matrix = 256 × 256, voxel resolution = 1.055 × 1.055 × 1.2 mm).

### 2.3 Image processing and analysis

Information on pre‐processing of the diffusion weighted images is available in the Supporting Information. All subject's FA images were aligned to the FMRIB_58 template in standard space, and a group mean FA skeleton was created (thresholded at 0.2). Each subject's FA data were then projected onto the group skeleton and FA values from the nearest relevant tract centre used to fill the subject‐specific FA skeleton. Voxel‐wise analyses were then carried out across all subjects on the skeletonised FA images.

A general linear model was implemented through FSL's Randomise (https://fsl.fmrib.ox.ac.uk/fsl/fslwiki/Randomise) at each voxel in the FA skeleton to examine group differences whilst controlling for scanning site, age, IQ and gender. Contrasts were specified to identify group differences as well as age‐by‐group interaction effects. A cluster‐based threshold was defined (Z > 2.3), and permutation testing was applied to obtain family‐wise error (FWE) corrected cluster *p*‐values (5000 Monte Carlo permutations). Results were thresholded at p_FWE_ < 0.05. This process was applied to the main analysis comparing AD with HC and to the subgroup analysis of alcohol‐only and alcohol‐polydrug groups. Locations of significant clusters were identified using the John Hopkins ICBM DTI‐81 probabilistic white matter atlas.^44^

### 2.4 Other statistical analysis

All other analyses and visualisations were carried out using R v3.6.3.^45^, ^46^, ^47^ Group differences in demographic variables were analysed using Welch's *t*‐test, analysis of variance (ANOVA) tests or Mann–Whitney *U*‐tests based on the distribution of data. Spearman's rank correlations and partial correlations were carried out to explore the relationship between FA from clusters identified through voxel‐wise analysis and clinical variables of interest. For clusters identified in the main analysis contrasting AD and HC, local FA was examined in relation to alcohol exposure, pack‐years and months abstinent from alcohol. Given a lack of differences between subgroups (alcohol‐only and alcohol‐polydrug), we explored associations between local FA and cannabis, cocaine and opiate exposure in the alcohol‐polydrug group. This was only examined in this group as the alcohol‐only group had very few individuals with exposure to these substances. We applied a false‐discovery rate correction to adjust for multiple comparisons (p_FDR_ < 0.05).^48^

## 3 RESULTS

### 3.1 Sample characteristics

Demographic information and patterns of drug use are provided in Table 1. The HC and AD groups were similar in terms of age, gender, site distribution and body mass index. The HC group had a higher estimated IQ than the AD group. As expected, the AD group had higher lifetime exposure to alcohol, cocaine, opiate, nicotine and cannabis as well as a higher proportion of current smokers, nicotine dependence and family history of dependence compared with HCs. Follow‐up data from telephone interviews was available for 34 AD participants, of which 12 (35%) had relapsed in the past 12 months since the study.

**TABLE 1:** Sample characteristics for each groups

Sample characteristics						
Demographics	Healthy controls (*n* = 65)	Alcohol dependent (*n* = 64)	Group comparisons	AD‐only, *n* = 28	AD‐poly, *n* = 36	Group comparisons
Age (years)	40 ± 17	43 ± 13	t_(126.65)_ = −1.68, *p* = 0.10	48 ± 12.0	41 ± 10.5	t_(58.81)_ = −2.48; *p* = 0.02
Age range (years)	21–64	25–64		30–60	25–64	
Gender ratio (M:F)	48:17	52:12	Χ^2^_(1)_= 0.63; *p* = 0.43	22:6	30:6	Χ^2^_(1)_ = 0.03; *p* = 0.87
WTAR IQ adjusted score	110 ± 9	104 ± 14	W = 2871.5; *p* < 0.001	105.5 ± 10.8	102.0 ± 14.5	W = 599.5; *p* = 0.20
Site of scan (Imperial:Camb:Man)	19:31:15	32:19:13	Χ^2^_(2)_ = 6.33; *p* = 0.04	14:8:6	18:11:7	Χ^2^_(2)_ = 0.05; *p* = 0.98
Years of education	15 ± 5	11 ± 2	W = 2926; *p* < 0.001	11 ± 2	11 ± 2	W = 551; *p* = 0.50
Family history of dependence (%)	5 (0.08%)^a^	29 (45%)	Χ^2^_(1)_ = 20.8; *p* < 0.001	9 (32%)	20 (56%)	Χ^2^_(1)_ = 2.6; *p* = 0.11
Body mass index	25.5 ± 3.84	25.8 ± 4.41	W = 1984; *p* = 0.76	24.9 ± 4.3	25.4 ± 4	W = 490; *p* = 0.86
Drug and alcohol dependence						
Lifetime cocaine dependence (%)		29 (45%)			29 (81%)	
Lifetime opiate dependence (%)		22 (34%)			22 (61%)	
Lifetime nicotine dependence (%)	35 (53%)	57 (89%)	Χ^2^_(1)_ = 22.7, *p* < 0.001	22 (79%)	35 (93%)	Χ^2^_(1)_ = 2.7, *p* = 0.10
Current smokers (%)	31 (48%)	47 (73%)	Χ^2^_(1)_ = 7.9; *p* = 0.005	20 (71%)	27 (75%)	Χ^2^_(1)_ = 0.001; *p* = 0.97
Lifetime drug and alcohol exposure^b^						
Alcohol exposure (years)	0 ± 0^c^	15 ± 11		19.5 ± 9	13.0 ± 12	
Normalised alcohol exposure		0.39 ± 0.27		0.41 ± 0.15	0.34 ± 0.17	t_(62)_ = −1.79; *p* = 0.08
Cocaine exposure (years)		1.5 ± 6		0 ± 0^c^	5 ± 5.88	
Normalised cocaine exposure		0.03 ± 0.16			0.12 ± 0.17	
Opiate exposure (years)		0 ± 6			5.5 ± 9.5	
Normalised opiate exposure		0 ± 0.14			0.11 ± 0.22	
Cannabis exposure (years)	0 ± 0^c^	3 ± 11		0 ± 0^c^	7 ± 10.5	
Normalised cannabis exposure		0.07 ± 0.25			0.21 ± 0.30	
Nicotine exposure (pack/year)	1.1 ± 20	20.7 ± 20	W = 876; *p* < 0.001	21.1 ± 19.4	20.4 ± 19.5	W = 427; *p* = 0.98
Abstinence length						
Months' abstinence (alcohol)		7.8 ± 21.4		6 ± 13.5	8 ± 21.2	W = 414, *p* = 0.30
Months' abstinence (most recent drug)		7.9 ± 20.6			9 ± 21.1	
Relapsed within 12 months (Yes:No)		12:22		7:10	5:12	Χ^2^_(1)_ = 0.13, *p* = 0.72
Age of regular use (for those with exposure)						
Alcohol	18 ± 3	15 ± 3	W = 2732, *p*<0.001	16 ± 3	15 ± 3.3	W = 654.5; *p* = 0.04
Cocaine		23 ± 8.5		34 ± 14^d^	22 ± 8	W = 110.5; *p* = 0.05
Opiates		20.5 ± 5.8			20.5 ± 5.8	

*Note*: Data are presented as median ± interquartile range.

^a^ Two missing values.

^b^ Exposure is given in years, and normalised exposure is calculated by dividing individual exposure by age, scaling the variable to range 0–1 with 1 being their entire lifetime.

^c^ The median and interquartile range were zero, but the data did contain non‐zero values.

^d^ Two individuals had a history of heavy cocaine use but no lifetime diagnosis of dependence.

In the comparison of alcohol‐only and alcohol‐polydrug, the alcohol‐polydrug group was younger and had a slightly lower mean IQ. As expected, cocaine, opiate and cannabis exposure were higher in the alcohol‐polydrug compared with alcohol‐only group, and age at regular use of alcohol was younger than for the alcohol‐only group. In contrast, alcohol exposure was higher in the alcohol‐only group compared with the alcohol‐polydrug group, though there was no difference when age was taken into account. They did not differ on current tobacco smoking status, nicotine dependence or lifetime nicotine exposure. Subgroups showed similar rates of relapse within 12 months following the study.

### 3.2 Whole‐brain analyses: Lower FA in AD compared with HC

**FIGURE 1:**
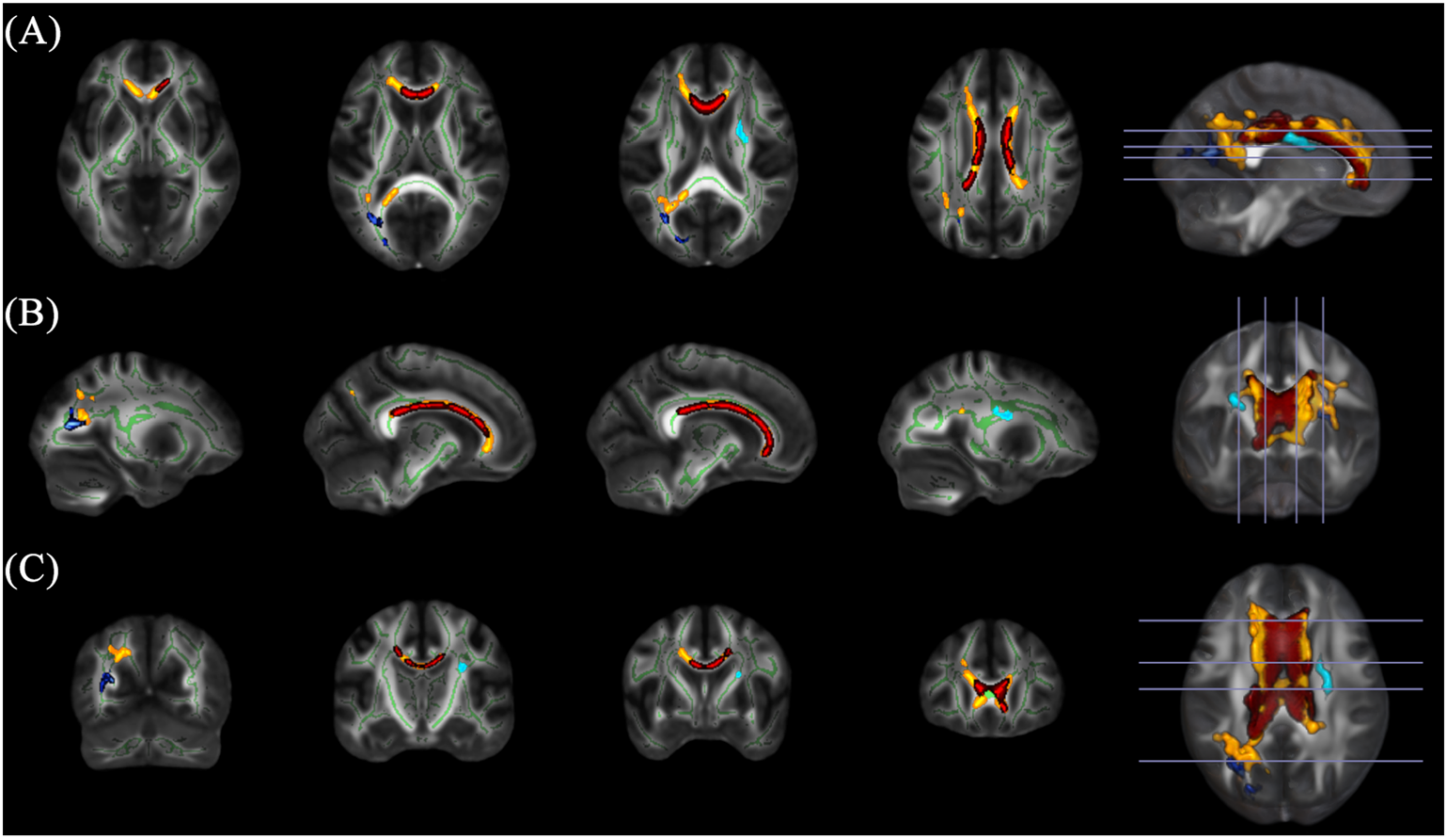
Lower FA in AD compared with HC shown across axial (A), sagittal (B) and coronal (C) slices. A main effect of group was found in the corpus callosum (shown in yellow, Cluster 1) as well as the anterior corona radiata (shown in light blue, Cluster 2). Group‐by‐age interaction effects, whereby a greater age‐related reduction was found in AD, were present in the corpus callosum (shown in red, Cluster 3) and in the posterior thalamic radiation (shown in dark blue, Cluster 4). The mean FA skeleton is shown in green. Clusters were inflated to improve visibility

**FIGURE 2:**
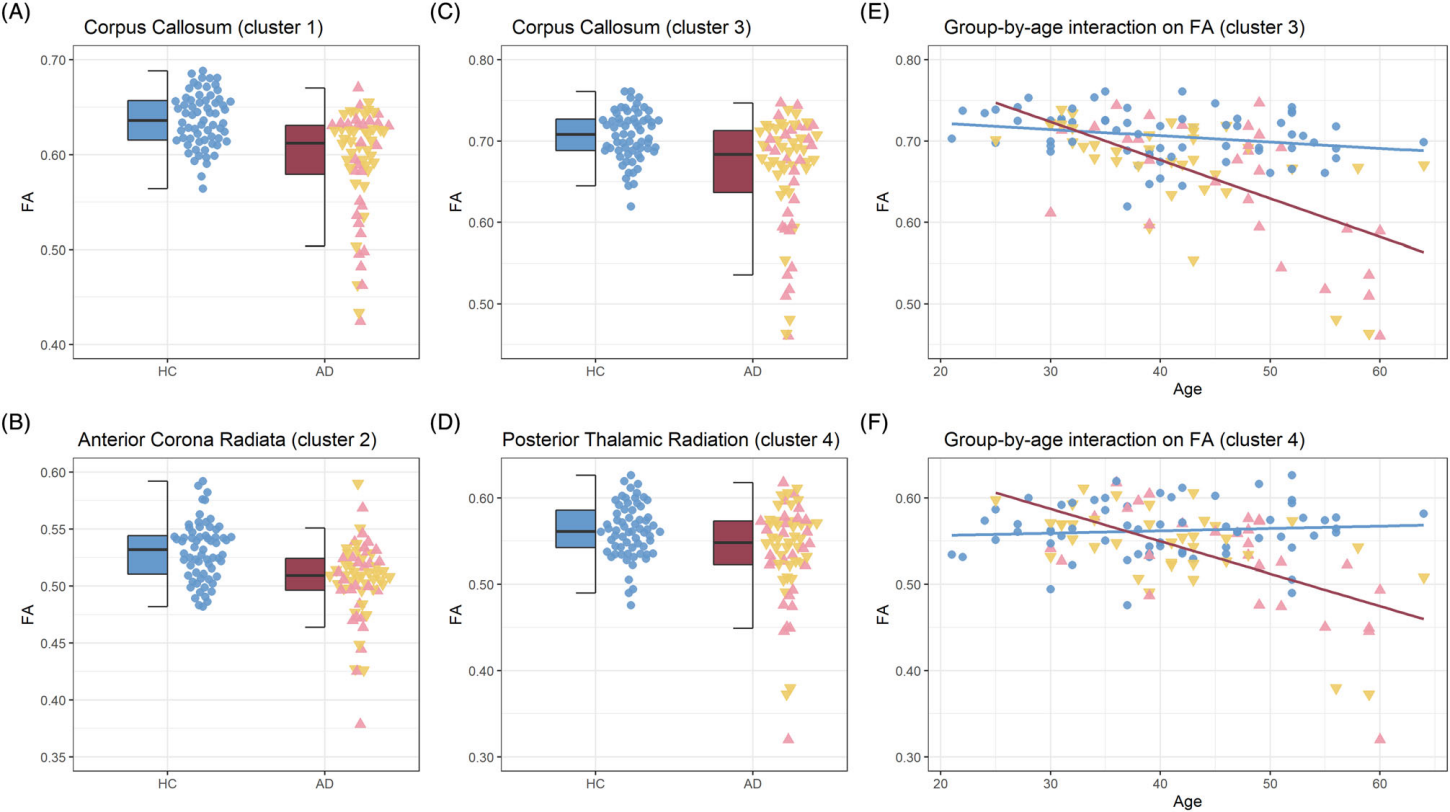
Mean FA values extracted from each cluster and plotted for each group (HC vs. AD). (A) A main effect of group across the corpus callosum where AD showed lower FA than HC. (B) A main effect of group in the right anterior corona radiata where AD showed lower FA than HC. (C,E) The group‐by‐age interaction effect on FA in the corpus callosum where those with AD showed greater reductions with age. (D,F) A similar group‐by‐age interaction effect in the left posterior thalamic radiation. HC are depicted in blue and AD in red. AD subgroups that were considered for further analysis are plotted using a different colour (alcohol‐only: pink upward‐facing triangles, alcohol‐polydrug: yellow downward‐facing triangles)

Whole‐brain analysis found significantly lower FA in AD relative to HC in a large cluster spanning the genu, body and splenium of the corpus callosum (Cluster 1) and a second smaller cluster in the right anterior corona radiata (Cluster 2). A group‐by‐age interaction effect was also found in two clusters, where a greater linear decrease in FA with age was found in AD. The larger cluster was found in the corpus callosum and showed considerable overlap with the group effect (Cluster 3). The smaller cluster was located in the left posterior thalamic radiation (Cluster 4). There were no clusters where FA was higher in AD. Cluster information is summarised in Table 2 and visualised in Figure 1. Mean FA was extracted from each cluster for each individual, and group and interaction effects are depicted in Figure 2.

**TABLE 2:** Summary of clusters highlighting differences between HC and AD

Cluster index	Effect	Cluster Size	Location	Cluster peak MNI coordinates			Cluster *p*‐value	Median cluster FA	
				X	Y	Z		Control	AD
1	Group	5815	Corpus callosum	11	28	−8	*p* < 0.001	0.64 ± 0.04	0.61 ± 0.05
2	Group	284	Right anterior corona radiata	31	−6	15	*p* = 0.047	0.53 ± 0.03	0.51 ± 0.03
3	Group‐by‐age	3174	Corpus callosum	13	32	−9	*p* < 0.001	0.71 ± 0.04	0.68 ± 0.08
4	Group‐by‐age	334	Left posterior thalamic radiation	−33	−64	6	*p* = 0.042	0.56 ± 0.04	0.55 ± 0.05

To further assess the laterality of effects reported in the right anterior corona radiata and left posterior thalamic radiation, we also examined group differences at a more lenient statistical threshold (p_FWE_ < 0.1). Lowering the threshold highlighted a similar group effect in the left anterior corona radiate whereby FA was lower in AD. There was no sign of an interaction effect in the right posterior thalamic radiation. Results are further reported in Table S2.

No significant differences in FA were found between alcohol‐only and alcohol‐polydrug groups.

### 3.3 Relationship between substance use and FA in AD

**FIGURE 3:**
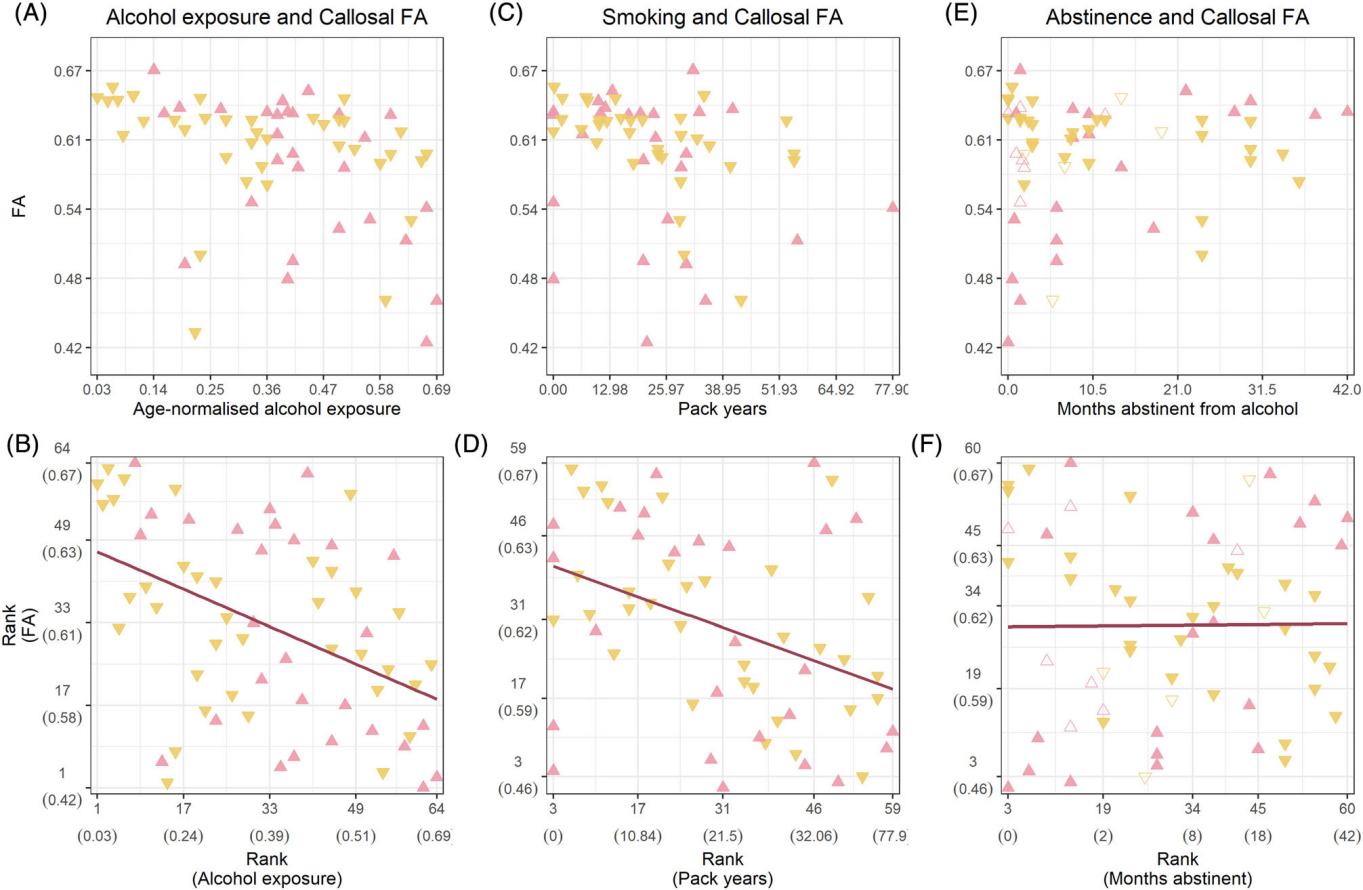
Associations between callosal FA and alcohol exposure and smoking in AD. Top: (A,C) A negative relationship between FA and alcohol exposure and FA and pack‐years, respectively. (E) No clear relationship between FA and months abstinent from alcohol. Bottom: Effects have been plotted using the ranked data to show the correlation strength. (B,D) A negative monotonic relationship was found between mean FA from Cluster 1 and alcohol exposure and pack‐years. (F) No clear relationship between FA and months abstinent from alcohol. AD subgroups that were considered for further analysis are plotted using a different colour (alcohol‐only: pink upward‐facing triangles, alcohol‐polydrug: yellow downward‐facing triangles). In (E) and (F), individuals who relapsed within 12 months are shown as open triangles

We sought to further examine group differences in FA by extracting the mean FA from the main cluster in the corpus callosum. A negative correlation was found between mean FA and age‐normalised alcohol exposure (ρ_(62)_ = −0.45, *p* < 0.001). Similarly, a negative correlation was found between smoking (pack‐years) and callosal FA (ρ_(57)_ = −0.39, *p* = 0.002). No correlation was found between FA and months abstinent from alcohol (ρ_(58)_ = 0.001, *p* = 0.94). We visually inspected relapse in relation to abstinence and FA, and though most individuals who relapsed had shorter lengths of abstinence at the time of the study, there was no distinguishable pattern in relation to callosal FA. Effects are plotted in Figure 3. Partial correlations showed that when controlling for smoking, alcohol exposure still showed a negative correlation with FA (ρ_(56)_ = −0.42, *p* = 0.001). The same was true for the negative correlation between smoking and FA whilst controlling for alcohol exposure (ρ_(56)_ = −0.31, *p* = 0.02). Examination within the alcohol‐polydrug group further showed no association between FA and age‐normalised cocaine (ρ_(34)_ = −0.12, *p* = 0.48), opiates (ρ_(34)_ = −0.05, *p* = 0.78) or cannabis exposure (ρ_(34)_ = 0.07, *p* = 0.70).

## 4 DISCUSSION

The purpose of this study was to investigate the relationship between AD and white matter microstructure as well as the association with measures of drug and alcohol consumption. In accordance with our hypothesis, we found that white matter FA was lower in AD relative to HC, particularly within corpus callosum. An interaction between age and group was identified where AD participants exhibited a greater decline in FA with age compared with HC. Furthermore, negative relationships with alcohol and nicotine exposure (pack‐years) were identified and shown to have independent effects on FA in AD. Contrary to our hypotheses, we found no differences in FA between alcohol‐only and alcohol‐polydrug participants, and exposure to substances such as cocaine or opiates had no discernible effect on FA. Surprisingly, duration of abstinence from alcohol did not correlate with FA. Overall, our findings show that FA is lower in AD and not further affected by additional substance dependences. Furthermore, the differences are more likely driven by lifetime exposure to alcohol and nicotine than by other polydrug dependence and do not appear to recover with abstinence. Lower FA may be additionally exacerbated by age‐related changes that appeared to be accelerated in the AD group.

In line with our primary hypothesis, we found lower FA in AD across the corpus callosum and extending into parts of the corona radiata. These findings are consistent with a recent meta‐analysis of white matter in AD highlighting convergent macro‐ and microstructural WM alterations in the corpus callosum.^13^ We demonstrated that callosal effects are negatively associated with long‐term excessive alcohol consumption. The anterior corpus callosum has been highlighted as a vulnerable region to the effects of alcohol, and a negative association with consumption has been shown in a cohort of adult alcohol drinkers.^49^ The potential mechanisms for changes to white matter in AD are numerous. Several studies of AD have examined additional diffusion metrics—mean, axial and radial diffusivity—thought to reflect different facets of white matter microstructure and have typically found higher radial diffusivity along with lower FA in the corpus callosum.^5^, ^8^, ^10^ Additionally, increases in these diffusivity indices have been reported in AD along commissural, associative and projection suggesting alterations beyond alterations to FA.^5^, ^12^, ^50^, ^51^ Whereas further confirmatory research is needed to, these studies suggest that though microstructural damage to the corpus callosum is a common feature of AD, there may be a more widespread pattern of alterations to white matter. Possible causes for these alterations include axonal damage and loss of myelin,^52^ though it is important to note that DTI alone is not sufficient to determine the mechanism underlying the alterations to white matter. Preclinical studies have shown that in vitro ethanol treatment can disrupt platelet‐derived growth factor receptor alpha (PDGFRα), which is required for the differentiation of oligodendrocyte progenitor cells (OPCs) into myelin‐producing oligodendrocytes.^53^ Additionally, chronic alcohol exposure has been found to activate Toll‐like receptor 4 (TLR4), which induces a cellular signalling cascade that leads to microglial activation. Microglia have been reported to release pro‐inflammatory factors that instigate an inflammatory response that can lead to axonal damage as well as damage to oligodendrocytes and to myelin binding proteins.^54^

In this study, we found a negative correlation between cigarette ‘pack‐years’ and FA. Furthermore, partial correlations showed that pack‐years and alcohol exposure each had an independent impact on callosal FA. One previous study found significant negative correlations between smoking duration and FA in the splenium of the corpus callosum.^17^ Another reported no effect of smoking status on FA in a mediation analysis,^55^ although this study was in heavy drinkers rather than AD and only considered recent smoking, rather than lifetime exposure.^55^ Our findings suggest that smoking does have additional detrimental effects on white matter microstructure. Previous literature has linked smoking severity and duration to lower FA, though this has been in non‐alcohol‐dependent smokers.^39^, ^56^ Further studies demonstrated that smoking history was a factor that affected FA recovery when comparing participants that were 1‐week abstinent with those 1‐month abstinent,^14^ and longer smoking duration was associated with lower FA in a cohort of alcohol‐dependent relapsers.^18^ Drinking and smoking behaviours commonly occur together, and it is therefore possible that an interaction effect between nicotine and alcohol exposure could contribute to these findings. It has previously been found that smoking AD had lower grey matter volume in the putamen than non‐smoking AD, but this study observed no effect on white matter volume.^57^ Our approach allowed us to consider the correlation between pack‐years and alcohol exposure in AD when examining their respective associations with FA, but our dataset was not suitable to further assess the possibility of an interaction. The possibility that nicotine exposure can affect white matter FA in conjunction with alcohol may also be clinically important considering that around 80% of alcohol‐dependent individuals smoke cigarettes,^58^ a figure also reflected in our sample.

We further explored the interaction effect between age and group and identified greater age‐related reductions in AD. This effect showed considerable spatial overlap with our main effect of group, suggesting that lower FA in AD is exacerbated with age and is likely associated with lifetime alcohol and nicotine consumption. In recent years, there has been renewed support for the notion of accelerated ageing in AD both in grey matter^59^, ^60^, ^61^ and in white matter.^15^, ^62^, ^63^ In particular, a greater age‐related decline in the corpus callosum was found in older compared with younger alcoholics.^62^ A subsequent TBSS study expanded on these findings and identified a similar interaction in the genu of the corpus callosum.^63^ A longitudinal assessment of FA in AD and controls did not find a greater reduction with age, except in those who had relapsed between scans.^30^ However, this study focused on trajectory slopes of FA for each individual, derived from regions showing a group difference, rather than through modelling the effect of age and AD on FA across the brain.

Our study found lower FA in AD, but there were no differences in FA between the alcohol‐only and alcohol‐polydrug subgroups. This was unexpected, as previous studies have shown effects of cocaine and opiate dependence on FA.^34^, ^35^, ^36^, ^37^, ^38^ Cocaine is a potent vasoconstrictor that has been associated with hypoperfusion and subsequent demyelination of neuronal cells.^64^, ^65^ It would therefore seem likely that cocaine has detrimental effects on white matter, but we are unable to support this with our data. Similarly, lower FA has been reported in opiate dependence.^37^, ^38^, ^66^, ^67^ Studies on polydrug use of other substances has produced mixed results. Lower FA in cocaine‐dependent polydrug users was not associated with alcohol use,^36^ and no differences were found between binge drinkers with and without heavy cannabis consumption.^68^ One study of cocaine users distinguished polydrug use subtypes—concurrent users of alcohol, cannabis or both—and found a negative association between FA and the number of additional substances used.^69^ However, subgroups were very small, and this study found no overall difference between controls and cocaine users. Overall, our findings do not point towards distinct effects of alcohol‐polydrug dependence on FA in abstinent individuals. Additionally, there was no indication of an association between lower FA and exposure to opiates, cocaine or cannabis, whereas alcohol and nicotine exposure showed a negative correlation with the observed group differences in FA.

Contrary to our hypothesis, we found no association between FA and length of abstinence in AD. Previous work has found evidence of possible recovery. A study comparing abstinence in AD reported increased callosal FA in those 1‐month abstinent compared with those 1‐week abstinent,^17^ and a longitudinal study of recently detoxified AD also demonstrated increased FA following protracted abstinence relative to baseline.^16^ In comparison, our study examined a younger cohort with a more mixed sample in terms of abstinence length. Furthermore, such studies have tended to assess the impact of only a single substance,^3^, ^28^, ^30^ whereas our cohort of alcohol‐dependent participants includes a significant number of individuals who are alcohol‐polydrug dependent. Such heterogeneity could mask the effects of a single substance on recovery. Indeed, cocaine and opiates are thought to exert distinct mechanisms of white matter dysregulation,^3^ which may require differing abstinence lengths in order for recovery to occur, or the effects may interact, resulting in no overall change in FA. In addition, different brain structures may be more or less susceptible to recovery. For example, amongst abstinent cocaine‐dependent individuals, some regional changes in FA persisted through abstinence, whereas others were altered as a function of abstinence, suggesting differential susceptibility to recovery.^70^ In addition to the possibility that the callosal FA is less susceptible to recovery, it may be that any recovery with abstinence is hindered by the exaggerated age‐related decline in AD. Due to high attrition rate during follow‐up, we were unable to formally investigate relapse, but visual inspection of the data did not show any sign of differences in callosal FA in those who relapsed within 12 months. Though abstinent from alcohol and illicit substances, the vast majority of AD participants were current smokers. This likely played a role in the observed differences in FA as well as the lack of abstinence‐related recovery; DTI studies of middle‐aged smokers have reported lower FA in the corpus callosum,^39^, ^40^ and have shown that smoking status and nicotine exposure can negatively affect recovery of FA with abstinence.^14^, ^17^, ^18^ Additionally, we found a negative association between callosal FA and pack‐years that was independent from the association with alcohol exposure. Taken together, it is possible that heavy nicotine consumption hinders expected recovery with abstinence from other substances.

The corpus callosum is a crucial white matter bundle that connects homologous structures in the two hemispheres, typically in a symmetric manner according to the topological organisation of the cortex.^71^, ^72^ As such, disruptions to myelination or axonal integrity along the corpus callosum can lead to a wide range of functional impairments. It is likely that these disturbances play a role in the cognitive profile seen in AD. Previous studies have found that alterations negatively affect executive function,^50^ working memory,^50^, ^62^ decision making,^7^ impulsivity^73^ and processing speed^12^, ^50^ and visuo‐spatial abilities.^62^ Disruptions to callosal white matter integrity in AD and the functional and clinical implications are worthy of further study, particularly if this is further exacerbated by age‐related decline^62^ and is non‐recoverable.

It is clear that caution should be exercised when generalising findings between one substance of dependence and another; certain regions may share commonality of effect regardless of dependence, whereas other changes may be substance‐specific or be subject to as yet unidentified interaction effects with other factors that could exacerbate and/or mitigate white matter alterations. Given the coincident use and abuse of substances (particularly cigarettes and alcohol) across one's lifetime, the possibility for disentangling the various impacts of single substances on white matter, and indeed brain structure more generally, is likely to pose a significant challenge. Coupled with evidence of differential susceptibility of regional changes to persistence versus recovery during abstinence further complicates the picture. Further investigation in longitudinal cohorts of much greater sample size and which include a range of individuals across cultures where patterns of concurrent use of certain substances may vary from those previously published will be required to understand the impacts of substances on brain structure more fully.

There are a few limitations that are relevant to the findings of this study. Firstly, the strength of interpretation is limited by its cross‐sectional design. We are unable to fully capture the impact of history of substance dependence or lifetime exposure to illicit substances when examining brain structure at a time where participants were already abstinent. Longitudinal designs allow greater confidence in linking changes in FA over time to exposure and abstinence, its relationship to the duration of active dependence and whether changes in FA impact the likelihood of relapse. Another limitation may be that our sample size was not sufficient to detect more subtle effects associated with additional substance dependences. Alcohol and nicotine have both been shown to have profound effects on white matter and may overlap with polydrug effects, making it harder to disentangle effects. Furthermore, population‐level studies are needed to understand the specific impact of substances such as cannabis, cocaine and opiates on white matter or the impact of age of onset, severity of dependence or whether the drugs were taken together or sequentially. Although effort was made to ensure healthy controls were age‐matched to the overall sample of dependent subjects, the alcohol‐only subgroup was slightly older than the alcohol‐polydrug subgroup. All groups were composed of a similar age range from 20 to 65 years old, and age was considered as a confounding variable in all our whole‐brain analyses. However, age was also of interest in relation to accelerated ageing, and we are unable to assert that group differences do not affect these results. Although only counting years of exposure when participants met the heavy use criteria produces a more reliable measure of exposure, it is not immune to ceiling effects whereby it is not possible to distinguish heavy and extreme levels of consumption. Furthermore, patterns of consumption may change over time, and our use of exposure is unable to fully consider differences in periods of drug use and abstinence. In terms of methodology, to build the mean FA skeleton, the participants' images were warped to a pre‐selected template rather than through building a template using the participants' own images. This template is an average image based on 58 well‐aligned good quality FA images from healthy male and female subjects that were between 20 and 50 years old. Variability in anatomy between individuals may be high, and template choice may impact the results in a group‐wise analysis.^74^ This may be because the detected group differences (from the statistical map) not only reflect the true group differences but also the degree of modification of each image to the template.^74^ If the participants' anatomy is close to that of the template, then less warping will occur, and the effect of template modification may be reduced. Again, such limitations can be addressed in larger sample sizes and with longitudinal designs that remove between‐subject variance.

## 5 CONCLUSIONS

This study contributes to our understanding of white matter microstructure in AD and offers novel insight into the role of polydrug use. Our findings indicate that deficits in white matter microstructure are present in AD and are associated with lifetime alcohol exposure. We further showed more pronounced age‐related reductions in FA in those with AD suggesting age‐related changes may be accelerated in this group. Furthermore, an independent association between nicotine exposure and FA was identified, but crucially, alcohol‐polydrug‐dependent individuals showed no additional differences in FA compared with alcohol‐only individuals. Further work is required to determine whether such alterations are associated with functional deficits and the extent to which these might be recoverable with treatment or longer‐term abstinence. With further validation, these alterations may provide useful biomarkers to predict the onset of problematic drinking, propensity to relapse or treatment response.

## CONFLICT OF INTEREST

David Nutt has been an advisor to British National Formulary, MRC, GMC and Dept of Health; is President of the European Brain Council; is past President of the British Neuroscience Association and European College of Neuropsychopharmacology; is chair of the Independent Scientific Committee on Drugs [UK]; is a member of the International Centre for Science in Drug Policy; is advisor to Swedish government on drug, alcohol and tobacco research; is editor of the *Journal of Psychopharmacology*; has sat on advisory boards at Lundbeck, MSD, Nalpharm, Orexigen, Shire, Awakn and COMPASSPathways; has received speaking honoraria (in addition to above) from BMS/Otsuka, GSK, Lilly, Janssen and Servier; is a member of the Lundbeck International Neuroscience Foundation; has received grants or clinical trial payments from P1vital, MRC, NHS and Lundbeck; has share options with P1vital; has been expert witness in a number of legal cases relating to psychotropic drugs; and has edited/written 27 books—some purchased by pharma companies. Trevor Robbins has held research grants with Eli Lilly and Lundbeck; has received royalties from Cambridge Cognition (CANTAB); has received editorial honoraria from Springer Verlag, Elsevier and Society for Neuroscience; has performed educational lectures for Merck, Sharpe and Dohme; and does consultancy work for Cambridge Cognition, Eli Lilly, Lundbeck, Teva and Shire Pharmaceuticals. Bill Deakin has carried out research funded by Autifony, Sunovion, Lundbeck, AstraZeneca and Servier. All payment is to the University of Manchester. Anne Lingford‐Hughes has received funding for research and/or PhD studentships from Alcarelle Ltd, Lundbeck and GSK; received honoraria (paid into University account) from Silence Therapeutics and NET Device Corp and consulted by but received no monies from Opiant, Camurus, Dobrin Consulting, Lightlake and GLG; received honoraria for talks and/or chairing from Janssen‐Cilag, Lundbeck and Servier; led the British Association for Psychopharmacology addiction guidelines (2012) that received support from Archimedes Pharma, Lundbeck, Pfizer and Schering. Louise Paterson has acted as research consultant (pharmacology) to Alcarelle Holdings Limited and received no payment. All other authors declare no conflict of interest.

## AUTHOR CONTRIBUTIONS

KA, LF and LP wrote the manuscript with support from ARLH. LP, LF and KA conceived of the presented idea and designed the experiment. LF and KA performed the analysis and LP supervised the analysis. LP, JM, RF, CO, AM, DGS and EMT were involved in data collection. RE, KDE, JS, TWR, JFWD, ARLH and DJN conceived of the overall ICCAM project and designed the overarching study. TWR, JFWD, DJN and ARLH obtained funding. All authors approved the manuscript.

## Supporting information




**Table S1.** Use of cocaine, opiates, and cannabis in each group for individuals with lifetime exposure for that substance. Exposure is given in years and normalised exposure is calculated by dividing individual exposure by age, scaling the variable to range 0‐1 with 1 being their entire lifetime.
**Table S2.** Summary of clusters highlighting differences between HC and AD when adopting a more lenient threshold (p_FWE_<0.1).

## Data Availability

The data that support the findings of this study are available on request from the corresponding author. The data are not publicly available due to privacy or ethical restrictions.

## References

[1] Monnig MA , Tonigan JS , Yeo RA , Thoma RJ , McCrady BS . White matter volume in alcohol use disorders: a meta‐analysis. Addict Biol. 2013;18(3):581‐592. 22458455 10.1111/j.1369-1600.2012.00441.x PMC3390447

[2] Pando‐Naude V , Toxto S , Fernandez‐Lozano S , Parsons CE , Alcauter S , Garza‐Villarreal EA . Gray and white matter morphology in substance use disorders: a neuroimaging systematic review and meta‐analysis. Transl Psychiatry. 2021;11(1):1‐18. 33431833 10.1038/s41398-020-01128-2 PMC7801701

[3] Hampton WH , Hanik IM , Olson IR . Substance abuse and white matter: findings, limitations, and future of diffusion tensor imaging research. Drug Alcohol Depend. 2019;197:288‐298. 30875650 10.1016/j.drugalcdep.2019.02.005 PMC6440853

[4] Smith SM , Jenkinson M , Johansen‐Berg H , et al. Tract‐based spatial statistics: voxelwise analysis of multi‐subject diffusion data. NeuroImage. 2006;31(4):1487‐1505. 16624579 10.1016/j.neuroimage.2006.02.024

[5] Yeh PH , Simpson K , Durazzo TC , Gazdzinski S , Meyerhoff DJ . Tract‐based spatial statistics (TBSS) of diffusion tensor imaging data in alcohol dependence: abnormalities of the motivational neurocircuitry. Psychiatry Res. 2009;173(1):22‐30. 19442492 10.1016/j.pscychresns.2008.07.012 PMC2774502

[6] Konrad A , Vucurevic G , Lorscheider M , et al. Broad disruption of brain white matter microstructure and relationship with neuropsychological performance in male patients with severe alcohol dependence. Alcohol Alcohol. 2012;47(2):118‐126. 22214998 10.1093/alcalc/agr157

[7] Zorlu N , Gelal F , Kuserli A , et al. Abnormal white matter integrity and decision‐making deficits in alcohol dependence. Psychiatry Res Neuroimaging. 2013;214(3):382‐388. 10.1016/j.pscychresns.2013.06.014 24080516

[8] Monnig MA , Caprihan A , Yeo RA , et al. Diffusion tensor imaging of white matter networks in individuals with current and remitted alcohol use disorders and comorbid conditions. Psychol Addict Behav. 2013;27(2):455‐465. 22352699 10.1037/a0027168 PMC3374918

[9] Sawyer KS , Maleki N , Papadimitriou G , Makris N , Oscar‐Berman M , Harris GJ . Cerebral white matter sex dimorphism in alcoholism: a diffusion tensor imaging study. Neuropsychopharmacology. 2018;43(9):1876‐1883. 29795404 10.1038/s41386-018-0089-6 PMC6046037

[10] De Santis S , Bach P , Pérez‐Cervera L , et al. Microstructural white matter alterations in men with alcohol use disorder and rats with excessive alcohol consumption during early abstinence. JAMA Psychiatry. 2019;76(7):749‐758. 30942831 10.1001/jamapsychiatry.2019.0318 PMC6583663

[11] Chumin EJ , Grecco GG , Dzemidzic M , et al. Alterations in white matter microstructure and connectivity in young adults with alcohol use disorder. Alcohol Clin Exp Res. 2019;43(6):1170‐1179. 30977902 10.1111/acer.14048 PMC6551253

[12] Galandra C , Crespi C , Basso G , et al. Decreased information processing speed and decision‐making performance in alcohol use disorder: combined neurostructural evidence from VBM and TBSS. Brain Imaging Behav. 2021;15(1):205‐215. 32124275 10.1007/s11682-019-00248-8

[13] Spindler C , Mallien L , Trautmann S , Alexander N , Muehlhan M . A coordinate‐based meta‐analysis of white matter alterations in patients with alcohol use disorder. Transl Psychiatry. 2022;12(1):1‐9. 35087021 10.1038/s41398-022-01809-0 PMC8795454

[14] Gazdzinski S , Durazzo TC , Mon A , Yeh PH , Meyerhoff DJ . Cerebral white matter recovery in abstinent alcoholics—a multimodality magnetic resonance study. Brain. 2010;133(4):1043‐1053. 20133395 10.1093/brain/awp343 PMC2850577

[15] Pfefferbaum A , Rosenbloom MJ , Chu W , et al. White matter microstructural recovery with abstinence and decline with relapse in alcohol dependence interacts with normal ageing: a controlled longitudinal DTI study. Lancet Psychiatry. 2014;1(3):202‐212. 26360732 10.1016/S2215-0366(14)70301-3 PMC4750405

[16] Alhassoon OM , Sorg SF , Taylor MJ , et al. Callosal white matter microstructural recovery in abstinent alcoholics: a longitudinal diffusion tensor imaging study. Alcohol Clin Exp Res. 2012;36(11):1922‐1931. 22551067 10.1111/j.1530-0277.2012.01808.x PMC3993083

[17] Zou Y , Murray DE , Durazzo TC , Schmidt TP , Murray TA , Meyerhoff DJ . Effects of abstinence and chronic cigarette smoking on white matter microstructure in alcohol dependence: diffusion tensor imaging at 4 T. Drug Alcohol Depend. 2017;175:42‐50. 28384535 10.1016/j.drugalcdep.2017.01.032 PMC5444327

[18] Zou Y , Murray DE , Durazzo TC , Schmidt TP , Murray TA , Meyerhoff DJ . White matter microstructural correlates of relapse in alcohol dependence. Psychiatry Res Neuroimaging. 2018;281:92‐100. 30273793 10.1016/j.pscychresns.2018.09.004 PMC6204088

[19] Lewohl JM , Wixey J , Harper CG , Dodd PR . Expression of MBP, PLP, MAG, CNP, and GFAP in the human alcoholic brain. Alcohol Clin Exp Res. 2005;29(9):1698‐1705. 16205370 10.1097/01.alc.0000179406.98868.59

[20] Hill SY , Terwilliger R , McDermott M . White matter microstructure, alcohol exposure, and familial risk for alcohol dependence. Psychiatry Res Neuroimaging. 2013;212(1):43‐53. 10.1016/j.pscychresns.2012.11.003 PMC3714312 23473988

[21] Jones SA , Nagel BJ . Altered frontostriatal white matter microstructure is associated with familial alcoholism and future binge drinking in adolescence. Neuropsychopharmacology. 2019;44(6):1076‐1083. 30636769 10.1038/s41386-019-0315-x PMC6461789

[22] Moss HB , Chen CM , Yi HY . Prospective follow‐up of empirically derived alcohol dependence subtypes in wave 2 of the National Epidemiologic Survey on Alcohol and Related Conditions (NESARC): recovery status, alcohol use disorders and diagnostic criteria, alcohol consumption behavior, health status, and treatment seeking. Alcohol Clin Exp Res. 2010;34(6):1073‐1083. 20374206 10.1111/j.1530-0277.2010.01183.x

[23] Quek LH , Chan GCK , White A , et al. Concurrent and simultaneous polydrug use: latent class analysis of an Australian nationally representative sample of young adults. Front Public Health [Internet]. 2013;1:61. doi: 10.3389/fpubh.2013.00061 PMC3860005 24350230

[24] Müller M , Ajdacic‐Gross V , Vetrella AB , et al. Subtypes of alcohol use disorder in the general population: a latent class analysis. Psychiatry Res. 2020;285:112712. 31837815 10.1016/j.psychres.2019.112712

[25] Staines GL , Magura S , Foote J , Deluca A , Kosanke N . Polysubstance use among alcoholics. J Addict Dis. 2001;20(4):57‐73. 10.1300/j069v20n04_06 11760926

[26] Kedia S , Sell MA , Relyea G . Mono‐ versus polydrug abuse patterns among publicly funded clients. Subst Abuse Treat Prev Policy. 2007;2(1):33‐41. 17996066 10.1186/1747-597X-2-33 PMC2211290

[27] Preti E , Prunas A , Ravera F , Madeddu F . Polydrug abuse and personality disorders in a sample of substance‐abusing inpatients. Ment Health Subst Use. 2011;4(3):256‐266.

[28] European Monitoring Centre for Drugs and Drug Addiction . Polydrug use: health and social responses [Internet]. 2021. Available from: https://www.emcdda.europa.eu/publications/mini-guides/polydrug-use-health-and-social-responses_en

[29] Grodin EN , Lin H , Durkee CA , Hommer DW , Momenan R . Deficits in cortical, diencephalic and midbrain gray matter in alcoholism measured by VBM: effects of co‐morbid substance abuse. NeuroImage Clin. 2013;2:469‐476. 24179800 10.1016/j.nicl.2013.03.013 PMC3777684

[30] Grodin EN , Momenan R . Decreased subcortical volumes in alcohol dependent individuals: effect of polysubstance use disorder. Addict Biol. 2017;22(5):1426‐1437. 27334243 10.1111/adb.12421 PMC5182196

[31] Pennington DL , Durazzo TC , Schmidt TP , Abé C , Mon A , Meyerhoff DJ . Alcohol use disorder with and without stimulant use: brain morphometry and its associations with cigarette smoking, cognition, and inhibitory control. PLoS ONE. 2015;10(3):e0122505. 25803861 10.1371/journal.pone.0122505 PMC4372577

[32] Mon A , Durazzo TC , Abe C , et al. Structural brain differences in alcohol‐dependent individuals with and without comorbid substance dependence. Drug Alcohol Depend. 2014;144:170‐177. 25263262 10.1016/j.drugalcdep.2014.09.010 PMC4280666

[33] O'Neill J , Cardenas VA , Meyerhoff DJ . Separate and interactive effects of cocaine and alcohol dependence on brain structures and metabolites: quantitative MRI and proton MR spectroscopic imaging. Addict Biol. 2001;6(4):347‐361. 11900613 10.1080/13556210020077073

[34] Lim KO , Wozniak JR , Mueller BA , et al. Brain macrostructural and microstructural abnormalities in cocaine dependence. Drug Alcohol Depend. 2008;92(1):164‐172. 17904770 10.1016/j.drugalcdep.2007.07.019 PMC2693223

[35] Ma L , Steinberg JL , Wang Q , et al. A preliminary longitudinal study of white matter alteration in cocaine use disorder subjects. Drug Alcohol Depend. 2017;173:39‐46. 28192722 10.1016/j.drugalcdep.2016.12.016 PMC5704923

[36] van Son D , Wiers RW , Catena A , Perez‐Garcia M , Verdejo‐García A . White matter disruptions in male cocaine polysubstance users: associations with severity of drug use and duration of abstinence. Drug Alcohol Depend. 2016;168:247‐254. 27736678 10.1016/j.drugalcdep.2016.09.023

[37] Wang Y , Li W , Li Q , Yang W , Zhu J , Wang W . White matter impairment in heroin addicts undergoing methadone maintenance treatment and prolonged abstinence: a preliminary DTI study. Neurosci Lett. 2011;494(1):49‐53. 21362458 10.1016/j.neulet.2011.02.053

[38] Wong NML , Cheung SH , Chan CCH , et al. Diffusivity of the uncinate fasciculus in heroin users relates to their levels of anxiety. Transl Psychiatry. 2015;5(4):e554. 25918991 10.1038/tp.2015.48 PMC4462611

[39] Lin F , Wu G , Zhu L , Lei H . Heavy smokers show abnormal microstructural integrity in the anterior corpus callosum: a diffusion tensor imaging study with tract‐based spatial statistics. Drug Alcohol Depend. 2013;129(1‐2):82‐87. 23062873 10.1016/j.drugalcdep.2012.09.013

[40] Savjani RR , Velasquez KM , Thompson‐Lake DGY , et al. Characterizing white matter changes in cigarette smokers via diffusion tensor imaging. Drug Alcohol Depend. 2014;145:134‐142. 25457737 10.1016/j.drugalcdep.2014.10.006

[41] Filbey FM , Aslan S , Calhoun VD , et al. Long‐term effects of marijuana use on the brain. Proc Natl Acad Sci. 2014;111(47):16913‐16918. 25385625 10.1073/pnas.1415297111 PMC4250161

[42] Gruber SA , Dahlgren MK , Sagar KA , Gönenç A , Lukas SE . Worth the wait: effects of age of onset of marijuana use on white matter and impulsivity. Psychopharmacology. 2014;231(8):1455‐1465. 24190588 10.1007/s00213-013-3326-z PMC3967072

[43] Paterson LM , Flechais RSA , Murphy A , et al. The Imperial College Cambridge Manchester (ICCAM) platform study: an experimental medicine platform for evaluating new drugs for relapse prevention in addiction. Part A: study description. J Psychopharmacol. 2015;29(9):943‐960. 26246443 10.1177/0269881115596155

[44] Mori S , Crain BJ . MRI Atlas of Human White Matter [Internet]. Amsterdam: Elsevier; 2006.

[45] R Core Team . R: a language and environment for statistical computing. R Foundation for Statistical Computing [Internet]. 2018. Available from: https://www.R-project.org/

[46] Wickham H . ggplot2: Elegant Graphics for Data Analysis. Seconded. Cham: Springer; 2016 260 p. (Use R!).

[47] Kim S . ppcor: an R package for a fast calculation to semi‐partial correlation coefficients. Commun Stat Appl Methods. 2015;22(6):665‐674. 26688802 10.5351/CSAM.2015.22.6.665 PMC4681537

[48] Benjamini Y , Hochberg Y . Controlling the false discovery rate: a practical and powerful approach to multiple testing. J R Stat Soc Ser B Methodol. 1995;57(1):289‐300.

[49] Topiwala A , Ebmeier KP , Maullin‐Sapey T , Nichols TE . No safe level of alcohol consumption for brain health: observational cohort study of 25,378 UK biobank participants [Internet]. bioRxvi. 2021. doi:10.1101/2021.05.10.21256931 PMC9163992 35653911

[50] Crespi C , Galandra C , Manera M , Basso G , Poggi P , Canessa N . Executive impairment in alcohol use disorder reflects structural changes in large‐scale brain networks: a joint independent component analysis on gray‐matter and white‐matter features. Front Psychol. 2019;10:2479. 32038340 10.3389/fpsyg.2019.02479 PMC6988803

[51] Crespi C , Galandra C , Canessa N , Manera M , Poggi P , Basso G . Microstructural damage of white‐matter tracts connecting large‐scale networks is related to impaired executive profile in alcohol use disorder. NeuroImage Clin. 2020;25:102141. 31927501 10.1016/j.nicl.2019.102141 PMC6953958

[52] Song SK , Sun SW , Ramsbottom MJ , Chang C , Russell J , Cross AH . Dysmyelination revealed through MRI as increased radial (but unchanged axial) diffusion of water. NeuroImage. 2002;17(3):1429‐1436. 12414282 10.1006/nimg.2002.1267

[53] Luo J , Miller MW . Basic fibroblast growth factor‐ and platelet‐derived growth factor‐mediated cell proliferation in B104 neuroblastoma cells: effect of ethanol on cell cycle kinetics. Brain Res. 1997;770(1):139‐150. 9372213 10.1016/s0006-8993(97)00762-2

[54] Alfonso‐Loeches S , Pascual M , Gómez‐Pinedo U , Pascual‐Lucas M , Renau‐Piqueras J , Guerri C . Toll‐like receptor 4 participates in the myelin disruptions associated with chronic alcohol abuse. Glia. 2012;60(6):948‐964. 22431236 10.1002/glia.22327

[55] Monnig MA , Yeo RA , Tonigan JS , et al. Associations of white matter microstructure with clinical and demographic characteristics in heavy drinkers. PLoS ONE. 2015;10(11):e0142042. 26529515 10.1371/journal.pone.0142042 PMC4631485

[56] Umene‐Nakano W , Yoshimura R , Kakeda S , et al. Abnormal white matter integrity in the corpus callosum among smokers: tract‐based spatial statistics. PLoS ONE. 2014;9(2):e87890. 24516568 10.1371/journal.pone.0087890 PMC3917830

[57] Durazzo TC , Mon A , Pennington D , Abé C , Gazdzinski S , Meyerhoff DJ . Interactive effects of chronic cigarette smoking and age on brain volumes in controls and alcohol‐dependent individuals in early abstinence: interactive effects. Addict Biol. 2014;19(1):132‐143. 22943795 10.1111/j.1369-1600.2012.00492.x PMC3528793

[58] Romberger DJ , Grant K . Alcohol consumption and smoking status: the role of smoking cessation. Biomed Pharmacother. 2004;58(2):77‐83. 14992787 10.1016/j.biopha.2003.12.002

[59] Thayer RE , Hagerty SL , Sabbineni A , Claus ED , Hutchison KE , Weiland BJ . Negative and interactive effects of sex, aging, and alcohol abuse on gray matter morphometry: sex, aging, alcohol abuse, and gray matter. Hum Brain Mapp. 2016;37(6):2276‐2292. 26947584 10.1002/hbm.23172 PMC4867295

[60] Guggenmos M , Schmack K , Sekutowicz M , et al. Quantitative neurobiological evidence for accelerated brain aging in alcohol dependence. Transl Psychiatry. 2017;7(12):1279. 29225356 10.1038/s41398-017-0037-y PMC5802586

[61] Sullivan EV , Zahr NM , Sassoon SA , et al. The role of aging, drug dependence, and hepatitis C comorbidity in alcoholism cortical compromise. JAMA Psychiatry. 2018;75(5):474‐483. 29541774 10.1001/jamapsychiatry.2018.0021 PMC5875381

[62] Pfefferbaum A , Adalsteinsson E , Sullivan EV . Dysmorphology and microstructural degradation of the corpus callosum: interaction of age and alcoholism. Neurobiol Aging. 2006;27(7):994‐1009. 15964101 10.1016/j.neurobiolaging.2005.05.007

[63] Sorg SF , Squeglia LM , Taylor MJ , Alhassoon OM , Delano‐Wood LM , Grant I . Effects of aging on frontal white matter microstructure in alcohol use disorder and associations with processing speed. J Stud Alcohol Drugs. 2015;76(2):296‐306. 25785805 10.15288/jsad.2015.76.296 PMC6463327

[64] Riezzo I , Fiore C , De Carlo D , et al. Side effects of cocaine abuse: multiorgan toxicity and pathological consequences. Curr Med Chem. 2012;19(33):5624‐5646. 22934772 10.2174/092986712803988893

[65] Bachi K , Mani V , Jeyachandran D , Fayad ZA , Goldstein RZ , Alia‐Klein N . Vascular disease in cocaine addiction. Atherosclerosis. 2017;262:154‐162. 28363516 10.1016/j.atherosclerosis.2017.03.019 PMC5757372

[66] Qiu Y , Jiang G , Su H , et al. Progressive white matter microstructure damage in male chronic heroin dependent individuals: a DTI and TBSS study. PLoS ONE. 2013;8(5):e63212. 23650554 10.1371/journal.pone.0063212 PMC3641135

[67] Bora E , Yücel M , Fornito A , et al. White matter microstructure in opiate addiction. Addict Biol. 2012;17(1):141‐148. 21070508 10.1111/j.1369-1600.2010.00266.x

[68] Jacobus J , McQueeny T , Bava S , et al. White matter integrity in adolescents with histories of marijuana use and binge drinking. Neurotoxicol Teratol. 2009;31(6):349‐355. 19631736 10.1016/j.ntt.2009.07.006 PMC2762024

[69] Kaag AM , van Wingen GA , Caan MWA , Homberg JR , van den Brink W , Reneman L . White matter alterations in cocaine users are negatively related to the number of additionally (ab)used substances. Addict Biol. 2017;22(4):1048‐1056. 26860848 10.1111/adb.12375

[70] Bell RP , Foxe JJ , Nierenberg J , Hoptman MJ , Garavan H . Assessing white matter integrity as a function of abstinence duration in former cocaine‐dependent individuals. Drug Alcohol Depend. 2010;114(2‐3):159‐168. 21075564 10.1016/j.drugalcdep.2010.10.001 PMC3062648

[71] Raybaud C . The corpus callosum, the other great forebrain commissures, and the septum pellucidum: anatomy, development, and malformation. Neuroradiology. 2010;52(6):447‐477. 20422408 10.1007/s00234-010-0696-3

[72] van der Knaap LJ , van der Ham IJM . How does the corpus callosum mediate interhemispheric transfer? A review. Behav Brain Res. 2011;223(1):211‐221. 21530590 10.1016/j.bbr.2011.04.018

[73] Liu IC , Chiu CH , Chen CJ , Kuo LW , Lo YC , Tseng WYI . The microstructural integrity of the corpus callosum and associated impulsivity in alcohol dependence: a tractography‐based segmentation study using diffusion spectrum imaging. Psychiatry Res Neuroimaging. 2010;184(2):128‐134. 10.1016/j.pscychresns.2010.07.002 20926265

[74] Shen S , Szameitat AJ , Sterr A . VBM lesion detection depends on the normalization template: a study using simulated atrophy. Magn Reson Imaging. 2007;25(10):1385‐1396. 17467945 10.1016/j.mri.2007.03.025

