# Supplementary material for: Alterations in white matter microstructure in alcohol and alcohol‐polydrug dependence: Associations with lifetime alcohol and nicotine exposure
Source: Addict Biol. 2022 Jul 18;27(5):e13207. doi: 10.1111/adb.13207 (PMC9540248; doi:10.1111/adb.13207)
Supplement: Supplementary file 1 — Table S1. Use of cocaine, opiates, and cannabis in each group for individuals with lifetime exposure for that substance. Exposure is given in years and normalised exposure is calculated by dividing individual exposure by age, scaling the variable to range 0‐1 with 1 being their entire lifetime. Table S2. Summary of clusters highlighting differences between HC and AD when adopting a more lenient threshold (pFWE<0.1). [file ADB-27-e13207-s001.docx]

**Exposure calculations**

Lifetime alcohol, cocaine, and opiate exposure and their nicotine equivalent pack years were defined as the number of years in which an individual’s alcohol or drug consumption was categorised as ‘heavy use’ for over 6 months within each 12-month period. ‘Heavy use’ of alcohol was defined when participants met daily and weekly high-risk drinking criteria (whichever was lower) according to World Health Organisation (WHO)^1^ and/or National Institute on Alcohol Abuse and Alcoholism (NIAAA)^2^ guidelines respectively, as follows:

Daily amounts of ≥ 8.75 UK units (≥ 70g of alcohol) for men and ≥ 7 UK units (≥ 56g of alcohol) for women^1^ were counted as alcohol exposure if drinking exceeded these limits for at least 3 days per week (i.e. binge-drinking behaviour). Weekly amounts were also counted if ≥ 50 UK units (~60g/day, 420 units total) for men, or ≥35 UK units (~40g/day, 280g total) for women^2^ were consumed (i.e. regular daily drinkers), whichever was lower. Since ‘high risk’ use of cocaine, opiate, and cannabis use is not well defined in the literature, the exposure criteria for these drugs were based upon clinical experience of ‘high risk’ use of these drugs. For cocaine exposure: > once per week of ≥1g per occasion. Opiate exposure was counted as > once per week of any amount. Finally, cannabis exposure was defined for those using daily or most days, at ≥2 spliffs per day. Exposure scores were normalised by dividing by the age of the participant.

**DTI data processing**

All data was pre-processed using Nipype^3,4^ ,a python interface, to allow a flexible workflow integrating neuroimaging packages. Denoising was carried out in Dipy through principal component analysis to remove components classified as noise based on the Marcenko-Pastur distribution using a 5x5x5 sliding window^5^ Gibbs-ringing artifacts were then removed through Dipy v1.3^6,7^. We corrected for motion and eddy currents using FSL 6.0.1^8^. Bias removal was done using ANTs v2.2^9^.on the b0 image and applied to the whole dwi-series. Distortion correction was carried out using a nonlinear registration-based approach with an inverted T_1_ image, previously processed using Freesurfer v6.0, as described in^10^. Data was upsampled to 1.5mm^3^ voxels using Dipy and the diffusion tensor was estimated using DTIFIT in FSL to produce maps of fractional anisotropy (FA).

**Supplementary Table 1.** Use of cocaine, opiates, and cannabis in each group for individuals with lifetime exposure for that substance. Exposure is given in years and normalised exposure is calculated by dividing individual exposure by age, scaling the variable to range 0-1 with 1 being their entire lifetime.

|  | Alcohol Dependent  (n=64) | Alcohol-only  (ADO, n=28) | Alcohol-plus  (AD+, n=36) |
| --- | --- | --- | --- |
| *Cocaine* |  |  |  |
| n (%) | 33 (52%) | 2 (7%) | 31 (86%) |
| Cocaine dependence (%) | 29 (45%) | 0 (0%) | 29 (81%) |
| Alcohol exposure | 14 ± 11 | 19 ± 4 | 13 ± 11 |
| Normalized alcohol exposure | 0.36 ± 0.28 | 0.45 ± 0.06 | 0.35 ± 0.28 |
| Cocaine exposure | 6 ± 5 | 3.5 ± 0.5 | 6.0 ± 5.5 |
| Normalised cocaine exposure | 0.15 ± 0.16 | 0.09 ± 0.02 | 0.17 ± 0.18 |
| Age regular use | 23 ± 8 | 31 ± 9 | 23 ± 8 |
| Months abstinent cocaine | 19 ± 32.5 | 18.9 ± 17.1 | 19 ± 34.3 |
| *Opiates* |  |  |  |
| n (%) | 22 (34%) |  | 22 (61%) |
| Opiate dependence (%) | 22 (34%) |  | 22 (61%) |
| Alcohol exposure | 11.8 ± 14.5 |  | 11.8 ± 14.5 |
| Normalized alcohol exposure | 0.26 ± 0.31 |  | 0.26 ± 0.31 |
| Opiate exposure | 8.5 ± 9 |  | 8.5 ± 9 |
| Normalised opiate exposure | 0.21 ± 0.23 |  | 0.21 ± 0.23 |
| Age regular use | 20.5 ± 5.75 |  | 20.5 ± 5.75 |
| Months abstinent opiates | 24 ± 83.5 |  | 24 ± 83.5 |
| *Cannabis* |  |  |  |
| n (%) | 36 (56%) | 6 (21%) | 30 (83%) |
| Alcohol exposure | 13 ± 9.5 | 15 ± 12 | 13 ± 8 |
| Normalized alcohol exposure | 0.34 ± 0.27 | 0.39 ± 0.17 | 0.34 ± 0.27 |
| Cannabis exposure | 10 ± 10 | 11 ± 2.25 | 9 ± 11.5 |
| Normalised cannabis exposure | 0.23 ± 0.26 | 0.22 ± 0.14 | 0.23 ± 0.27 |

**Supplementary Table 2.** Summary of clusters highlighting differences between HC and AD when adopting a more lenient threshold (p_FWE_<0.1).

| Cluster index | Effect | Cluster Size | Location | Cluster Peak  MNI Coordinates | | | Cluster  p-value |
| --- | --- | --- | --- | --- | --- | --- | --- |
|  |  |  |  | X | Y | Z |  |
| 1 | Group | 5647 | Corpus Callosum | 11 | 28 | -8 | p<0.001 |
| 2 | Group | 284 | Right Anterior Corona Radiata | 31 | -6 | 15 | p=0.049 |
| 3 | Group | 272 | Superior Frontal Gyrus | -15 | 47 | 22 | p=0.054 |
| 4 | Group | 230 | Left Anterior Corona Radiata | -18 | 5 | 12 | p=0.076 |
| 5 | Group-by-age | 3129 | Corpus Callosum | 13 | 32 | -9 | p=0.001 |
| 6 | Group-by-age | 531 | Left Posterior Thalamic Radiation | -33 | -64 | 6 | p=0.019 |
| 7 | Group-by-age | 284 | Right Anterior Corona Radiata | 23 | 35 | 2 | p=0.059 |

**References**

1. National Institute on Alcohol Abuse and Alcoholism. NIAAA Council Approves Definition of Binge Drinking. NIAAA Newsletter. 2004; Available from: https://pubs.niaaa.nih.gov/publications/Newsletter/winter2004/Newsletter_Number3.pdf

2. World Health Organization. In: SpringerReference. Berlin/Heidelberg: Springer-Verlag; 2011. Available from: http://www.springerreference.com/index/doi/10.1007/SpringerReference_301104

3. Gorgolewski K, Burns CD, Madison C, Clark D, Halchenko YO, Waskom ML, et al. Nipype: A Flexible, Lightweight and Extensible Neuroimaging Data Processing Framework in Python. Front Neuroinformatics. 2011;5.

4. Nipype. Available from: https://nipy.org

5. Veraart J, Fieremans E, Novikov DS. Diffusion MRI noise mapping using random matrix theory: Diffusion MRI Noise Mapping. Magn Reson Med. 2016 Nov;76(5):1582–93.

6. Kellner E, Dhital B, Kiselev VG, Reisert M. Gibbs-ringing artifact removal based on local subvoxel-shifts: Gibbs-Ringing Artifact Removal. Magn Reson Med. 2016 Nov;76(5):1574–81.

7. Neto Henriques R. Advanced Methods for Diffusion MRI Data Analysis and their Application to the Healthy Ageing Brain. 2017 Sep 28

8. Andersson JLR, Sotiropoulos SN. An integrated approach to correction for off-resonance effects and subject movement in diffusion MR imaging. NeuroImage. 2016 Jan;125:1063–78.

9. Tustison NJ, Avants BB, Cook PA, Zheng Y, Egan A, Yushkevich PA, et al. N4ITK: improved N3 bias correction. IEEE Trans Med Imaging. 2010 Jun;29(6):1310–20.

10. Wang S, Peterson DJ, Gatenby JC, Li W, Grabowski TJ, Madhyastha TM. Evaluation of Field Map and Nonlinear Registration Methods for Correction of Susceptibility Artifacts in Diffusion MRI. Front Neuroinformatics. 2017 Feb 21
